# Comparative Genomics of *Aeschynomene* Symbionts: Insights into the Ecological Lifestyle of Nod-Independent Photosynthetic Bradyrhizobia

**DOI:** 10.3390/genes3010035

**Published:** 2011-12-21

**Authors:** Damien Mornico, Lucie Miché, Gilles Béna, Nico Nouwen, André Verméglio, David Vallenet, Alexander A.T. Smith, Eric Giraud, Claudine Médigue, Lionel Moulin

**Affiliations:** 1 IRD-LSTM, UMR113, Campus de Baillarguet, 34398 Montpellier cedex 5, France; E-Mails: dmornico@genoscope.cns.fr (D.M.); lucie.miche@univ-provence.fr (L.M.); gilles.bena@ird.fr (G.B.); nico.nouwen@ird.fr (N.N.); eric.giraud@ird.fr (E.G.); 2 LABGeM, CEA-Genoscope & CNRS-UMR8030, 91057 Evry, France; E-Mails: vallenet@genoscope.cns.fr (D.V.); asmith@genoscope.cns.fr (A.A.T.S.); cmedigue@genoscope.cns.fr (C.M.); 3 LMBM, Faculté des Sciences, Université Mohammed V-Agdal, Av. Ibn Batouta BP 1014, Rabat, Morocco; 4 Laboratoire de Bioénergétique Cellulaire, CEA Cadarache, DSV, IBEB, 13108 Saint-Paul-lez-Durance, France; E-Mail: andre.vermeglio@cea.fr

**Keywords:** rhizobia, genomics, *Bradyrhizobium*, *Aeschynomene*, nod-independent

## Abstract

Tropical aquatic species of the legume genus *Aeschynomene* are stem- and root-nodulated by bradyrhizobia strains that exhibit atypical features such as photosynthetic capacities or the use of a *nod* gene-dependent (ND) or a *nod* gene-independent (NI) pathway to enter into symbiosis with legumes. In this study we used a comparative genomics approach on nine *Aeschynomene* symbionts representative of their phylogenetic diversity. We produced draft genomes of bradyrhizobial strains representing different phenotypes: five NI photosynthetic strains (STM3809, ORS375, STM3847, STM4509 and STM4523) in addition to the previously sequenced ORS278 and BTAi1 genomes, one photosynthetic strain ORS285 hosting both ND and NI symbiotic systems, and one NI non-photosynthetic strain (STM3843). Comparative genomics allowed us to infer the core, pan and dispensable genomes of *Aeschynomene* bradyrhizobia, and to detect specific genes and their location in Genomic Islands (GI). Specific gene sets linked to photosynthetic and NI/ND abilities were identified, and are currently being studied in functional analyses.

## 1. Introduction

Rhizobia are soil bacteria able to develop nitrogen-fixing symbioses with legumes, by the formation of organ-like structures on plant roots called nodules. Following differentiation into bacteroids, they transform atmospheric dinitrogen into ammonium that they provide to the plant in exchange for carbohydrates. Rhizobia are polyphyletic and spread among the classes Alphaproteobacteria and Betaproteobacteria [1]. *Bradyrhizobium* is a genus within alphaproteobacteria comprising many symbiotic species phylogenetically spread into three main clades: the *B. japonicum* clade, the *B. elkanii* clade and the photosynthetic *Bradyrhizobium* (PB) clade [2,3].

**Figure 1:**
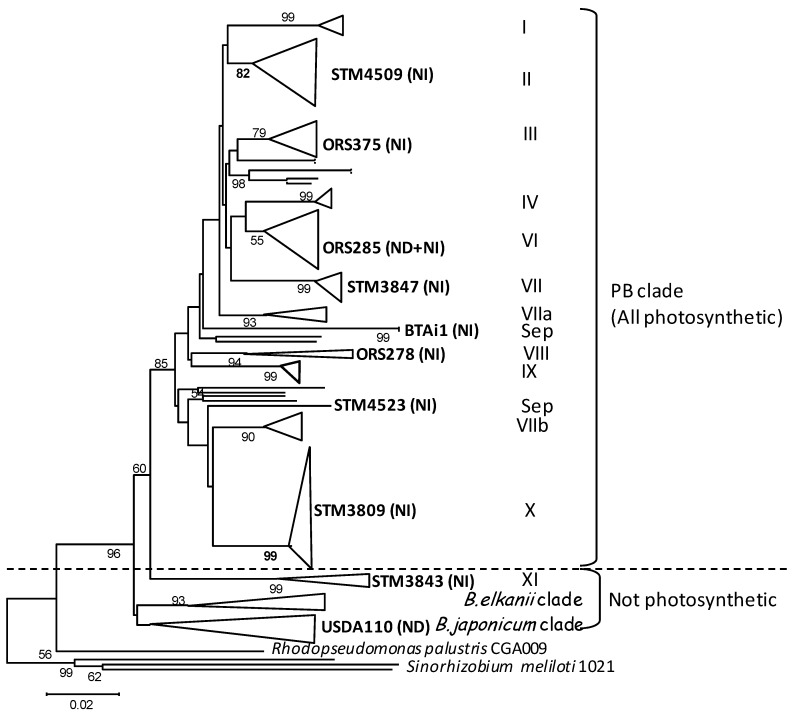
Maximum likelihood *recA* phylogeny of *Aeschynomene* bradyrhizobial symbionts indicating the position of genome-sequenced strains. Adapted from [16]. Numbers at nodes are bootstrap percentages from 100 replicates. Roman numbers indicate clades defined from AFLP analyses and *recA* phylogeny in [16]. Abbreviations: PB: photosynthetic bradyrhizobia; Sep: separate group with no affiliated number; ND/NI: nod gene dependent/independent symbiotic pathway.

The photosynthetic bradyrhizobia clade is composed of symbionts specific to the tropical *Aeschynomene* legume species, on which they form root but also stem nodules. Stem nodulation is a plant-dependent ability quite unusual in legumes, described in only five genera (*Aeschynomene*, *Discolobium*, *Neptunia*, *Sesbania* and *Vigna*) and is often associated to the plants’ aquatic life in wet or temporarily flooded habitats [4,5]*.* The infection process in *Sesbania rostrata* and *Aeschynomene* species during water-stress conditions occurs by “crack entry” (a process different from root hair curling) by intercellular infection of epidermal fissures (cracks) generated by the emergence of lateral roots [6,7]. The nodulation process between *Sesbania rostrata* and its specific symbiont *Azorhizobium caulinodans* occurs via a classic Nod-factor dependent process [7,8]. The symbiotic interaction between *Aeschynomene* and photosynthetic *Bradyrhizobium*, however, presents several particularities. First, by definition, PB possess a photosynthetic activity [9] that might play a role in symbiosis, either by conferring a selective advantage for bacterial survival *ex planta* (as a supplementary source of energy), and/or by facilitating bacterial infectivity and symbiotic effectiveness [10,11]. Second, some photosynthetic bradyrhizobia lack the canonical nodulation genes (genome-based analyses on ORS278 and BTAi1) and use an unknown *nod* gene-independent pathway to form nodules on some *Aeschynomene* species [12,13]. *Aeschynomene* species are categorized into three cross-inoculation (CI) groups [14,15], linked to specific features of their symbionts mentioned above: their photosynthetic ability, their capacity to form stem nodules, and finally their use of a *nod* gene-dependent (ND) or *nod* gene-independent (NI) symbiotic pathway. The CI group I includes *Aeschynomene* species nodulated only on their roots by non-photosynthetic bradyrhizobia, stem nodulation being restricted to CI group 2 and 3. CI group 3 is characterized by plant species nodulated only by PB lacking nodulation (*nod*) genes (NI, as strains ORS278 and BTAi1 symbiotic of *A. indica* and *A. sensitiva*), while CI group 2 species are nodulated by both photosynthetic and non-photosynthetic strains (as *A. afraspera*) [14]. It is relevant to note that photosynthetic strains able to nodulate CI group 2 (as ORS285) nodulate also CI group 3, as they carry both the ND and NI systems. Indeed an isogenic *nod*A minus mutant of ORS285 lost its symbiotic ability on CI group 2 but was still able to nodulate efficiently CI group 3 [13]. In a recent study, Miché and colleagues [16] sampled many rhizobial strains on CI group 3 *Aeschynomene* species in Central and South America. All isolates belonged to the PB clade, except a new group of non-photosynthetic strains phylogenetically placed at an intermediate position between the photosynthetic clade and the *B. japonicum*/*B. elkanii* clades, and nodulating the *Aeschynomene* CI group 3 (stem and roots) by the NI pathway (see Figure 1, STM3843 clade).

Research is currently under way to identify the genetic bases of the NI symbiotic pathway. Traditional genetic approaches using a random Tn5 transposon insertion allowed the identification of mutants affected in nodule development and nitrogen fixation [12]. However, no complete nodulation deficient mutants were identified during this screening, underlining a potential functional redundancy in genes involved in the early steps of this interaction, and the need for different approaches to identify them.

To better understand how photosynthetic bradyrhizobia have adapted to their particular niches (aquatic, nodules on *Aeschynomene*), we developed in this study a comparative genomics approach on several strains with a focus on the nod-dependent/nod-independent and photosynthetic/non-photosynthetic bacterial lifestyles. To reach this aim, we sequenced and compared the genomes of (i) five nod gene independent (NI) PB strains of CI group 3 from different sub-branches of the PB clade (ORS375, STM3809, STM3847, STM4509 and STM4523) and included the previously sequenced ORS278 and BTAi1 genomes; (ii) a PB strain hosting both ND and NI genetic bases (ORS285, CI group 2); (iii) a NI non-photosynthetic strain of CI group 3 (STM3843). Comparative genomics analyses allowed us to (i) infer the genomic diversity and evolutionary dynamics of PB genomes (core and accessory gene sets, Genomic Islands); (ii) identify gene sets potentially involved in symbiosis and photosynthesis. The latter are currently being studied by functional analyses.

## 2. Results and Discussion

### 2.1. Genomic Features of Bradyrhizobial Genomes

This study compares 10 *Bradyrhizobium* genomes, including nine genomes of *Aeschynomene* symbionts, and the genome of *B. japonicum* USDA110. Three complete genomes were already published (*Bradyrhizobium* sp. BTAi1, *Bradyrhizobium* sp. ORS278 [13], *B. japonicum* USDA110 [17]). Seven strains were sequenced for this study, selected as representatives of almost every phylogenetic branch of the photosynthetic bradyrhizobia clade (isolated from worldwide *Aeschynomene* species) as established in the *recA* gene phylogeny published in [16] (see Figure 1 for phylogenetic placement). Genetic features and sequencing methodologies used for each strain are given in Table 1.

**Table 1:** Bacterial strains and genome sequencing statistics.

Strain/Replicon	Group *	CI Group	Geographical Origin	ND/NI	Sequencing Status	Nb Contigs	Size (bp)	Accession Number
Photosynthetic *Bradyrhizobium* clade								
ORS278	VIII	3	Senegal	NI	Complete	1	7456587	NC_009445
BTAi1 Chr	Sep	3	USA	NI	Complete	1	8264689	NC_009485
BTAi1 Pl					Complete	1	228826	NC_009475
**ORS375**	III	3	Senegal	NI	454 + Solexa	497	7909110	CAFI01000001-497 ^&^
**STM3809**	X	3	F. Guiana	NI	454 + Solexa	803	7391986	CAFJ01000001-803 ^&^
**ORS285**	VI	2	Senegal	Both ^%^	454 (30X)	301	7632258	CAFH01000001-301 ^&^
**STM3847**	VII	3	F. Guiana	NI	Solexa 20X	152475	10121035 ^§^	ERP000868 ^$^
**STM4509**	II	3	Mexico	NI	Solexa 20X	190139	9206235 ^§^	ERP000868 ^$^
**STM4523**	Sep	3	Mexico	NI	Solexa 20X	158371	8610503 ^§^	ERP000868 ^$^
Non-photosynthetic strain								
**STM3843**	XI	3	F. Guiana	NI	454 + Solexa	350	8469730	CAFK01000001-350 ^&^
*B. japonicum*								
USDA110	Sep	1	USA	ND	Complete	1	9105828	NC_004463

Strain genomes sequenced in this study are in bold. * Species or AFLP group was defined according to [16]; Sep: separate group with no affiliated number; ND/NI: nod-dependent or nod-independent strain according to [13,16]; CI: *Aeschynomene* cross inoculation group according to [14,15]; ^%^: ORS285 hosts both systems; ^§^: genome size estimates from Solexa read contig assemblies; ^&^ EBI accession numbers; ^$^ ENA (trace archive) accession number for solexa reads [18].

Presence of plasmids was checked for by PFGE analysis (not shown), as well as the search for replication genes (*rep*ABC) within each genome. All 10 genomes seem to be composed of a unique chromosome, except strain BTAi1 which also harbors a plasmid [19]. *B. japonicum* USDA110 possesses the biggest genome with a length of 9,105,828 bp, and *Bradyrhizobium*. sp. STM3809 the smallest with 7,391,986 bp (Table 1; when excluding Solexa draft genomes for which size inference is uncertain). Bradyrhizobial genomes are GC rich bacteria with an average content of 65% (G + C). The protein coding density has an average of 86% and the number of CDS fluctuates between 6,750 (ORS278) and 9,650 (*B. japonicum*). Strains host between 3 and 6 rRNA genes in 1 to 3 operons, and 47 to 52 tRNA genes were identified in the various strains (Table 2). Codon and amino-acid usage is identical within bradyrhizobia (Supplementary Figure S1), which can be explained by the same GC genome content (64%–66%) and their phylogenetic proximity.

**Table 2:** Genomic features of bradyrhizobial genomes.

Strain	GC%	CDS N	CDS L	IGR (bp)	PCD (%)	rRNA	tRNA	MscRNA	NRR (%)
ORS278	65.51	6748	952.1	180.23	85.50	6	50	10	8.76
BTAi1 chr	64.92	7466	959.42	176.72	85.59	6	52	12	9.33
BTAi1 pl	60.71	257	800.2	186.02	79.42	-	-	-	4.75
ORS285	65.23	6848	955.92	184.32	85.41	4	49	11	10.45
ORS375	65.49	7348	921.28	182.92	84.99	3	52	11	10.22
STM3809	66.18	7142	879.79	182.97	84.19	3	47	13	9.42
STM3843	63.3	8399	878.57	159.54	86.30	3	48	10	5.10
BjUSDA110	64.06	9648	862.74	135.17	88.98	3	50	3	9.53

Abbreviations used: CDS: Coding Sequences; CDS-N: CDS number; CDS L: mean CDS Length; PCD: Protein coding density; Msc: Miscellanous; NRR: Nosferatu Repeated Region; bp: base pair.

### 2.2. Core, Dispensable and Strain-Specific Genes

**Figure 2:**
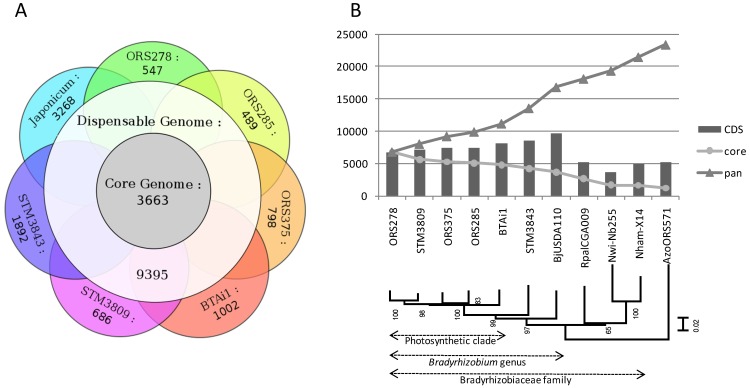
(**A**) Comparative gene orthology between seven genomes of *Bradyrhizobium*. Numbers indicates the shared gene sets between groups of strains, satisfying the following criteria: BBH (bidirectional best hits) with 40% amino-acid identity on 80% of the smallest protein; (**B**) Core and pangenome gene count in the Bradyrhizobiaceae family. CDS indicates the number of protein-coding genes in each genome listed in x-axis. The y-axis indicates the number of genes in core and pangenome when adding genomes in the x-axis to the comparative analysis. The presented tree was built by Maximum likelihood (GTR + I + G model) on a partition of 5 taxonomic marker genes (*atpD*, *dnaK*, *recA*, *rpoB*, *rpoD*), with bootstrapped nodes (100 replicates). Japonicum/Bj: *B. japonicum* USDA110; Rpal: *Rhodopseudomonas palustris*; Nwi: *Nitrobacter winogradskii*; Nham: *Nitrobacter hamburgensis*; Azo: *Azorhizobium caulinodans*.

A comparative analysis of gene content was undertaken to classify genes into groups depending on their degree of orthology/conservation among strains, following a bidirectional best hits approach (see Experimental Section). This analysis was performed on seven genomes (fragmented Solexa genomes were not included). First, genes present in strictly all seven genomes were pooled in a group called “core-genome”. Then, genes identified in only one genome were pooled in groups “specific” to each strain. All other genes, present in a least two genomes (but not in all), were pooled in a group called “dispensable genome”. Finally, the pangenome includes all genes (core, dispensable and specific). This analysis is illustrated by a Venn diagram presented in Figure 2A.

The core genome of bradyrhizobia (*i.e*., genes shared by all seven strains) is composed of 3,663 genes, of which 67% have an annotated function, 32% encode conserved proteins of unknown function, and only 1% are hypothetical proteins (see Supplementary Figure S2A). This core gene set included all 206 genes from the minimal bacterial gene-set described in [20]: genes encoding DNA replication or repair machinery (DNA polymerase subunits, gyrase or helicase), transcription and translation enzymes and factors (RNA polymerase, amino-acids-tRNA synthases, ribosomal proteins, *etc*.), transport (PTS system), energy and intermediary metabolism (ATP synthase, acyl-CoA synthase, glycolysis enzymes). The dispensable genome was the largest group with 9,395 genes, reflecting a high rate of horizontal gene transfer and the metabolic versatility among bradyrhizobia. It was composed of 8% of hypothetical proteins, 35% of conserved proteins of unknown function, and 65% of proteins with functional annotation (statistics almost identical to the core genome annotation). The specific gene set of each genome ranges from 489 to 3,268 genes, and was directly related to the phylogenetic distance between one strain and its closest neighbors. For example, a strong correlation was found between the phylogenetic distance of *Bradyrhizobium* strains towards ORS278 (based on a distance matrix of the 6 genes used in MLSA for Figure 2B) and their number of specific genes compared to ORS278 (R^2^ = 0.87). *B. japonicum* USDA110 is the most distant from other strains (harboring 3,268 specific genes) followed by STM3843 (1,892 specific genes), that is at an intermediate phylogenetic distance between USDA110 and the photosynthetic strains clade (Figure 2B).

Photosynthetic strains harbors between 489 and 1,002 specific genes. BTAi1 genome exhibits the largest number of specific genes, of which 125 are located on its plasmid (pBRAB). pBRAB contains 296 CDS, meaning that 171 (57%) of these plasmidic genes are chromosomal in other strains, but they were not arranged in groups with obvious synteny to the chromosomes. Among the strain-specific gene sets, most genes were annotated as hypothetical protein (from 37% to 66% depending on strain) or conserved protein of unknown function (4% to 27%; with orthologs founds in RefSeq or Uniprot databases, see Supplementary Figure S2A–C), but several functions of ecological interest could be identified. For example, the ORS278 specific gene set encodes gas vesicle proteins (used by cyanobacteria to adjust their position in water depending on light conditions [21]) and RTX toxins (gram-negative exotoxin); the BTAi1 one encodes transporters involved in heavy metal resistance, a type IV secretion system, uptake hydrogenases (Hup/Hyp proteins, recycling of hydrogen produced during the nitrogen fixation process in legume nodules, [19]), a siderophore, an additional ATP synthase; the ORS285 specific gene set encodes several non-ribosomal peptide synthetase, a glycopeptide antibiotic (Tyrocidine synthetase 3); ORS375 contains specific genes encoding enzymes involved in anaerobic benzoate degradation pathway (AliB), gas vesicle proteins (GvpF/L), arsenic resistance; a bacteriophytochrome (BphB); STM3809 harbors specific genes encoding enzymes involved in catechol (CatABCD) and aromatic acid degradation (XylY, XylZ, BenA, BenD). In the case of STM3843 (1,892 specific genes), it was possible to identify functions involved in vitamin biosynthesis (B1, B2, B12), fumarate catabolism, urease metabolism, many adenylate/guanylate cyclases (regulation of AMPc/GMPc secondary messengers), rhizopine catabolism (*moc* genes), fermentation of carbohydrates (pyruvate pathway-*por*AB genes), biosynthesis of LPS and EPS (*exo* genes, *O*-antigen synthesis), and several toxin/antitoxin (TA, as YoeB-YefM) modules involved in bacterial stress response and/or stabilization of chromosomal integrons [22].

Each group of genes was functionally annotated according to the KEGG classification and distribution of functional categories of genes were compared between strain-specific, dispensable and bradyrhizobial core genes (shown in Supplementary Figure S2B,C). In general, specific and dispensable gene sets exhibited the same KEGG distribution, both differing highly in proportions from the core genome for several functions such as “xenobiotics degradation and metabolism” and “membrane transport” (mainly ABC transporters and secretions systems; see Supplementary Figure S2B) that are over-represented in the specific and dispensable gene sets, or cell routine functions (translation, protein folding, nucleotide metabolism) that are almost specific to the core gene set.

A distribution of the core and pangenome at different taxonomic levels (PB clade, *Bradyrhizobium* genus, Bradyrhizobiaceae family) is shown in Figure 2B. The core genome of the PB clade (including 5 photosynthetic bradyrhizobia) is composed of 4,792 genes, which decreases to 4,208 for *Aeschynomene* symbionts (PB clade + STM3843; excluding Bj USDA110), then to 3,663 (*Bradyrhizobium* genus), 1,632 (Bradyrhizobiaceae family, including *Rhodopseudomonas* and *Nitrobacter* genomes), and finally drops to 1,212 after inclusion of an out-family alphaproteobacteria (*Azorhizobium*). The pangenome gene number increases dramatically at each addition of a new bacterial genome, reaching 12,040 genes at 5 PB strains, with an increased rate when shifting taxonomical borders (13,898 with STM3843, 16,488 with USDA110, ending at 20,162 for the Bradyrhizobiaceae family). It does not seem to reach a plateau even after addition of 5 PB genomes, reflecting the large genomic diversity within this clade. By comparison, the core and pangenome of 5 *Rhodopseudomonas* strains (Bradyrhizobiaceae family, closely related to bradyrhizobia and belonging to at least 4 several species) contain 3,319 and 8,000 genes, respectively [23], around 30% less than the *Bradyrhizobium* genus.

### 2.3. Whole Genome Comparative Analyses and Distribution of Genomic Islands

**Figure 3:**
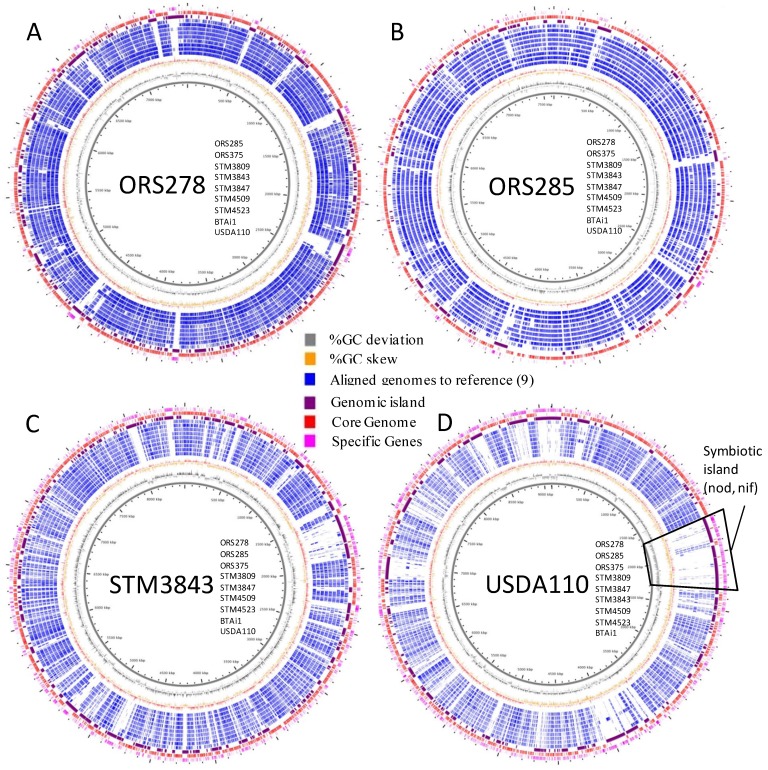
Circular view of whole genome alignments of bradyrhizobial genomes. Genomes were aligned to a reference genome [for (**A**) ORS278, (**B**) ORS285, (**C**) STM3843, (**D**) USDA110)]. The localization of aligned genomes is given as a list from inner to outter circles in the center of each figure. Legend (from inner to outter layer of circles: genome scale (kb), GC deviation (gray), GC skew (orange), aligned genomes (aligned parts are in blue, each layer is a different genome, strains are listed close to the reference genome name in the center of each figure, from inner to outer layers), GI (purple), core genome (red), specific genes to reference genomes (pink). Higher resolution images for each genome analysis are available in Supplementary Figure S4.

Whole genome alignments were performed on the basis of a reference genome (see Experimental Section). Figure 3 presents whole genome alignments based on ORS278 (3A), ORS285 (3B), STM3843 (3C) and USDA110 (3D) with 9 genomes of *Bradyrhizobium* (other genomes and larger genome views are available as Supplementary Figure S4). This representation allows inclusion of complete, draft and even highly fragmented genomes (obtained by Solexa technology).

The first result obtained from Figure 3 is the high genome homology between strains from the PB clade (darker blue layers on Figure 3A,B), while less apparent when STM3843 (Figure 3C) or USDA110 (Figure 3D) were used as reference genomes (lighter blue circles around the reference genome). Interestingly, when *B. japonicum* USDA110 was used as the reference genome (Figure 3D), the 680 kb symbiotic island (localized between 1.7 and 2.3 Mb in USDA110 genome, integrated into a val-tRNA gene) did not show any match with the *Aeschynomene* symbionts genomes, except for *nif* and *fix* genes for all strains (encoding the symbiotic nitrogen fixation system), the *nodABCIJ* and a type 3 secretion system for ORS285 (that use both ND and NI systems). However, circular views also allow the detection of various “small” GI, exemplified by holes in the blue layers around the reference genome. As shown in Figure 3, many GI were found among genomes, some being shared between strains. This analysis was further explored using RGP finder (see Experimental Section), and all detected GI were numbered and their distribution among bradyrhizobial genomes was investigated (except on the 3 solexa-based genomes). Supplementary Table S1 presents the listing and distribution of 613 detected GI (see Mat&Methods section for details), with associated functions and distribution in bradyrhizobial genomes. In PB genomes (5 genome-based), 278 GI were found (53 to 78 GI per genome), of which 33 (11%) host phage-related functions, 14 (6%) carry conjugal transfer genes, and 45 (16%) carry transposases or fragments of transposases. Overall, these GI carrying mobile elements represents 27% of PB GI-hosted genes.

**Figure 4:**
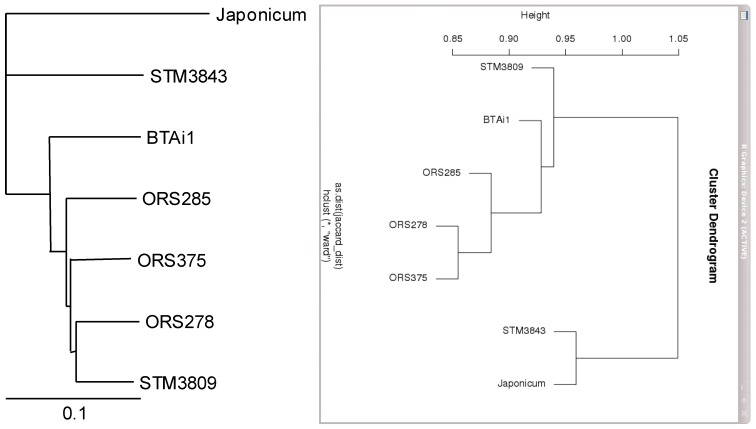
Comparison of core-genome based phylogeny and a dendrogram based on GI distribution across bradyrhizobial genomes.

A dendrogram based on the presence/absence of GI was built, and compared to a phylogeny based on the core genome set (Figure 4). Interestingly, both trees were slightly discordant with a different position of STM3809 within the PB clade that seemed correlated in the GI-based dendrogram with the geographical origin of strains (STM3809 and BTAi1 are of American origin, while ORS strains are of West African origin). In both trees, STM3843 and *B. japonicum* USDA110 diverged from the PB clade, reflecting their high taxonomic distance to the PB clade.

### 2.4. Metabolic Network Comparison of Strains

**Figure 5:**
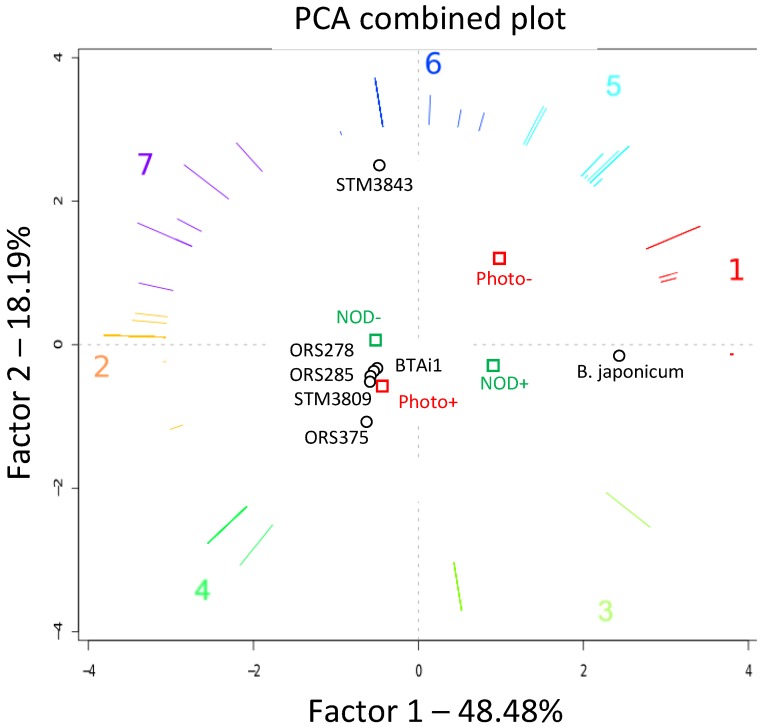
Principal Component Analysis (PCA) of seven Bradyrhizobium genomes, performed on a two dimensional matrix compiling metabolic pathway completion values (the number of enzymatic reactions with coding genes for pathway x in a given organism, divided by the total number of reactions in pathway x as defined in the MetaCyc database; see Experimental Section) across all genomes. In the plot, each genome is represented by a point, whereas pathways are shown as colored vectors. Pathways with correlated completion values across organisms (vectors with similar orientation) have been clustered (corresponding to numbers 1 to 7) and drawn in the same color (vectors of pathways with identical completions overlap). Genomes can be associated with their representative and characteristic groups of metabolic pathways (*i.e*., vectors “pointing in their direction”). The corresponding pathway functions are listed in Supplementary Table S2. Vector length encodes the quality of representation of the pathway in the presented plot (*i.e*., the longer a vector is, the more representative its pathway is for genomes on the same side of the plot). Abbreviations: photo−/photo+: non-photosynthetic/photosynthetic strains; Nod+/Nod−: nod-dependent/nod-independent strains).

A principal component analysis was performed to describe the completion (measured as a percentage) of all known metabolic pathways present in the seven *Bradyrhizobium* genomes in order to find metabolic specificities or common pathways among several strains. This analysis was associated with the symbiotic and photosynthetic phenotypes of strains. The three first factorial axis (Figure 5, Supplementary Figure S5) captured over 80% of the total data variability. The first factor (Factor 1) isolates metabolic specificities of *B. japonicum* USDA110 against other strains, Factor 2 captures variability of STM3843 compared with the others, and the third axis (Factor 3) separates BTAi1 from the other genomes.

Strain segregation in the PCA plots can be explained by the metabolic differences which are larger in USDA110, STM3843 and BTAi1 compared to other strains. On the first factorial plane (Figure 5), 64 pathways more complete in *B. japonicum* are represented by red vectors pointing in its direction, indicative of many metabolic specificities of this strain compared to others (biosynthesis of various molecules from carbohydrates to amino-acids, degradation of many xenobiotics, see Supplementary Table S2). Pathways more complete in STM3843 (separated from others on second factorial plane, vector group 6, 20 pathways) included metabolic enzymes involved in biosynthesis and degradation of various substrates. Specific metabolic pathways for photosynthetic strains (vector 4) included 13 pathways with known roles in photosynthesis (bacteriochlorophyll a biosynthesis, phytyl diphosphate biosynthesis). Finally, 60 pathways more complete in nod-independent strains (First factorial plan, vector 2 and 7) separated them from nod-dependent ones. These pathways included many degradation enzymes (of molecules such as atrazine, nitrobenzene, purine, pyrimidine) and biosynthesis enzymes (for products as phycocyanobilin).

### 2.5. Functional Exploration of Genomes: Focus on Photosynthetic and Symbiotic Abilities

**Figure 6:**
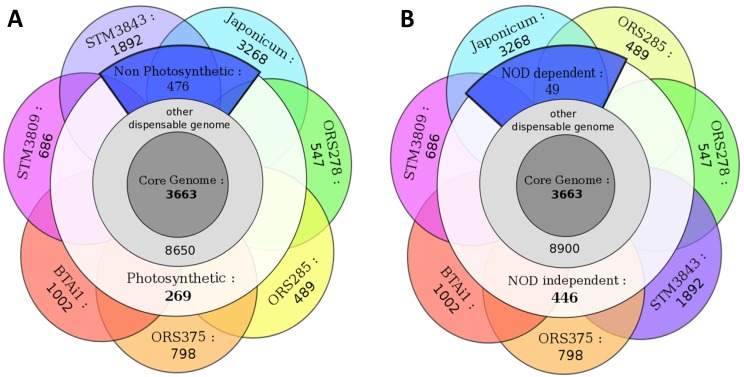
Phyloprofile exploration of genomes according to photosynthetic (**A**) and nodulation (**B**) abilities.

Orthologs within dispensable gene groups were searched for among strains sharing the photosynthetic/non-photosynthetic and nod-dependent/nod-independent phenotypes (strain using *nod* genes to develop their symbiosis with the plant host or not). The phylogenetic profile (“phyloprofile” for short) exploration of genomes is presented in Figure 6A (photosynthesis) and Figure 6B (symbiosis). A KEGG functional classification of specific genes for each phenotype is presented in Supplementary Figure S3.

#### 2.5.1. Photosynthetic *Versus* Non-Photosynthetic Strains

The specific gene set of PB is composed of 269 genes, of which 50% were annotated with functions known to be involved in bacterial photosynthesis (see Supplementary Table S3 for the complete listing of genes), 64 (23%) were annotated as hypothetical proteins, and 74 (27%) as conserved proteins of unknown functions. Supplementary Table S4 presents a list of photosynthetic strain-specific gene set with annotated functions, sort by functional roles.

Bacterial photosynthesis has been shown to play an important role during stem symbiosis with *Aeschynomene* plants. It has been proposed that during the early steps of symbiosis, the energy provided by the photosynthetic apparatus facilitates *ex planta* survival and infectivity whereas during the later steps this energy can be used by the bacteria to fix nitrogen, thus limiting the demand for plant photosynthetates [10]. All the genes necessary for the formation of the photosystem (PS) are clustered in a 50 kb-region, the so-called “photosynthesis gene cluster” or PGC that includes genes found in the 269 specific gene set of PB: (i) *bch* genes involved in bacteriochlorophyll biosynthesis, (ii) *crt* genes involved in carotenoid biosynthesis (spirilloxanthin), (iii) *puc* and *puf* genes which encode respectively the core proteins of the reaction center (RC) and for the light-harvesting complexes I (LHI), and (iv) several regulators (bacteriophytochrome and *ppsR* genes). The organization of the genes in the PGC is perfectly conserved in all the photosynthetic *Bradyrhizobium* strains examined during this study (ORS278, BTAi1, ORS285, ORS375 and STM3809) whereas none of these genes can be found in the non-photosynthetic *Bradyrhizobium* strains (USDA110 and STM3843). It is hypothesized that the ancestor of the *Bradyrhizobium japonicum* and *B. elkanii* clades was a photosynthetic free-living bacteria that later adapted to soil conditions and acquired nodulation genes, leading subsequently to a loss of the photosynthetic character due to a light-devoided habitat [11]. The absence of any hallmark of acquisition of the PGC by lateral gene transfer (absence of insertion sequences, GC% nearly identical to the whole genome, no GC skew deviation), a phylogeny of *pufL* being nearly identical to the one of the core genome (not shown), and the fact that *Bradyrhizobium* species are closely related with the photosynthetic *Rhodopseudomonas palustris* species sustain this hypothesis.

The synthesis of the photosynthetic apparatus is under the control of an original regulatory mechanism that has been described in ORS278 and BTAi1 strains [24,25]. Its formation occurs only under far-red light illumination and semi-aerobic conditions and involves a control by a light receptor (the bacteriophytochrome *Br*BphP1) and two transcriptional factors of the PpsR family (PpsR1 which is redox sensitive acts as an activator whereas PpsR2 acts as a repressor and its activity is modulated by *Br*BphP1). These three regulators are encountered in the three other photosynthetic *Bradyrhizobium* strains, suggesting that this unusual mechanism of regulation of PS synthesis by light and oxygen tension is a typical feature of this bacterial group. This regulatory circuit appears to be perfectly adapted to promote PS synthesis during stem symbiosis [24].

Unlike ORS278 strain, the PS apparatus of BTAi1 strain contains additional peripheral light-harvesting complex (LH2) encoded by a *pucBA* operon. The expression of this operon is also under the control of a bacteriophytochrome (*BBta_3080*) found at the vicinity of the *pucBA* operon [26]. Interestingly, this gene organization is found in two of the other three photosynthetic strains (ORS375 and STM3809) that were studied, suggesting that the presence of LH2 complexes is rather frequent in PB strains.

Several bacteriophytochromes genes have been detected in photosynthetic *Bradyrhizobium* strains (2 to 4). In particular, one bacteriophytochrome (BRADO2008) is encountered in all PB as well as in the non-photosynthetic strain STM3843. Up to now, no function for this bacteriophytochrome has been identified and no genes in relation with light could be found at its vicinity. We suspect that this function is not in relation with photosynthesis as it is also found in the non-photosynthetic strain STM3843 and no alteration in the photosynthetic activity of an ORS278 mutant was detected (data not shown). Some PB strains possess specific bacteriophytochromes. The ORS278 strain contains the gene BRADO1262, located on a Horizontally Acquired Island (HAI) together with genes involved in phycocyanobilin (PCB) and gas vesicle syntheses [27]. This bacteriophytochrome binds PCB as a chromophore instead of biliverdin and displays very atypical spectral properties [27]. The ORS375 strain also possesses a specific bacteriophytochrome (BRAO375v1_4770021) hosted on a HAI, though no functional data are yet available for this bacteriophytochrome.

The relatively high number of bacteriophytochromes found in PB strains (2 to 4, while 1 in STM3843, and none in *B. japonicum* USDA110) indicate that these bacteria are well equipped to cope with a luminous environment, which is probably related to their ability to occupy an ecological niche exposed to sunlight (aquatic environment and phyllosphere). This niche is different from those of “classic” rhizobia, which inhabit soil and usually lack any bacteriophytochromes.

Photosynthesis can be defined as the biological process by which organisms make carbohydrates from CO_2_ using energy captured from sunlight. The functional relationship between the photosystem and the Calvin Benson Bassham cycle (CBB) is also found at the genetic level. Indeed in all PB strains studied, a *cbb* gene cluster is found contiguous to the PGC. Interestingly, whereas the PGC has been lost (or is absent) in the non-photosynthetic strains (USDA110 and STM3843), a *cbb* gene cluster is found in perfect synteny in photosynthetic strains. These *cbb* genes are surely functional as it has been clearly demonstrated that *B. japonicum* is able to fix CO_2_ in presence of Hydrogen [28]. The maintenance of a functional CBB cycle might be the result of a selective advantage but its role remains unknown. Recently, it has been described in the ORS278 strain that Rubisco, the key enzyme of the Calvin cycle, is required for an efficient symbiosis with *A. indica* [29]. It has been proposed that the primary role of the CBB cycle is not to produce biomass but to act as an electron sink driving the cell’s excess electrons to CO_2_ and thus oxidizing reduced factors. This role might be critical in the early steps of symbiosis when oxygen tension in the nodule becomes limiting and the nitrogenase activity is not yet established. In such conditions, the functioning of the CBB cycle might help deal with the pool of reduced cofactors produced by the Krebs cycle.

#### 2.5.2. Focus On Nod-Dependent (ND) and Nod-Independent (NI) Symbiotic Strains

Since our genome set included strains exhibiting ND and NI symbiotic phenotypes, we looked at specific gene sets present only in nod-independent strains, by subtracting the *B. japonicum* USDA110 gene set from the core genome of all *Aeschynomene* symbionts (ORS278, ORS285, BTAi1, ORS375 and STM3809). We hypothesized that all NI strains possess conserved genetic bases to establish their symbiotic interaction. This analysis revealed 446 genes specific to NI strains (Figure 6B). This relatively high number of genes is probably related to the close phylogenetic distance between the PB strains (thus sharing many metabolic pathways due to their common ecological niche) and their monophyly. This gene set was also explored in solexa-based draft genomes (STM3847, STM4509 and STM4523) using tblastn searches, and 62 genes were found absent among the 3 genomes, though their absence could be the consequence of the fact that these genomes aren’t completely assembled. These absent genes encoded mainly hypothetical or conserved hypothetical proteins.

The functional annotation of the 446 genes (see Supplementary Figure S3 for KEGG classification and Supplementary Table S5 for a complete listing) showed 104 hypothetical proteins (23%), 125 conserved proteins of unknown function (28%), and 217 (49%) genes with annotated functions of which 23 had a gene name. Several functions putatively involved in symbiosis were found and are listed in Table 3:

(i) Regulatory genes of which many are two-component regulatory proteins (9 genes with sensor/histidine kinase domains), diguanylate cyclase (BRADO0188, BRADO1402, BRADO6510), circadian clock proteins KaiB and KaiC involved in circadian rythms (BRADO1478 and 1479);
(ii) Catabolic enzymes: catabolic pathways for protocatechuate (BRADO2235 to 2343, BRADO2378-2379) and vanillate degradation (BRADO1851), two compounds derived from lignin monomers [30] (lignin being an abundant constituent of plant cell walls); NUDIX hydrolases that hydrolyse a wide range of organic pyrophosphates and reflect the metabolic complexity and adaptability of a given organism [31]; various catabolic enzymes especially in Taurine degradation;
(iii) *N*-acetyl-glucosamine modifying enzymes as UDP-*N*-Acetylglucosamine 1-carboxyvinyltransferase (BRADO6639), with some proteins containing TPR repeats (BRADO4235, BRADO7123);
(iv) Polysaccharide & glycan biosynthesis with 2 specific NI gene regions: BRADO4794-4814 with enzymes involved in wall techoic acid biosynthesis, an anionic polymer that decorate the peptidoglycan layers of many Gram-positive organisms; and BRADO5154-5204 located on a GI in all PB strains;
(v) Biosynthesis of various molecules including carotenoids (BRADO0659), Indole-3-acetic acid (IAA) (BRADO7016), antibiotics (BRADO1346), cobalamin (B12 vitamin, BRADO4898-4917), and a type 3 polyketide synthase (BRADO0151);
(vi) Nitrogen fixation as *nifV* (BRADO5390), encoding an homocitrate synthase responsible for *ex planta* N_2_ fixation ability [32], as well as several nitrogen-fixation related genes (BRADO5414, 5416, 5418, 5424) clustered in an operon conserved among all NI strains as well as in *Azorhizobium caulinodans* ORS571, but not in rhizobia as *B. japonicum* USDA110, *S. melioti* 1021 or *M. loti* MAFF303099 (that do not fix nitrogen in the free-living state). Interestingly, this gene cluster is up-regulated in *A. caulinodans* ORS571 bacteroids on *Sesbania rostrata* stem nodules, indicating a probable specific role in symbiotic nitrogen fixation in these symbionts;
(vii) Carbon monoxide oxidation (BRADO6025-6032), that catalyzes the reversible conversion between carbon dioxide (CO_2_) and carbon monoxide (CO), and found in many marine bacterial genomes;
(viii) Chemotaxis: several methyl-accepting chemotaxis proteins (receptor/sensory transducer);
(ix) LuxI-LuxR quorum sensing with BRADO0941 involved in the biosynthesis of a cinnamoyl-HSL (Homo Serine Lactone), an unusual aryl-HSL [33];
(x) Transport: General secretion pathway protein (BRADO6338-type II, BRADO6341, 6344), HlyD-family protein (BRADO7122).

Some NI candidate genes were mutated by directed Tn5 insertions (BRADO7016-IAA biosynthesis, BRADO0151-type 3 polyketide synthase, and BRADO3025-1,4-alpha-glucan branching enzyme) but none of the obtained mutants were found to be altered in their symbiotic performance suggesting that these determinants are not critical for the Nod-independent process [34].

Localization in GI was also investigated for the NI specific gene set. We had anticipated that the NI symbiotic pathway genes could be carried by a genomic island conserved among the NI strains. No signature of a conserved large symbiotic island (as in the USDA110 SI that harbored 60% of transposases of the whole genome [17]) could be identified among the NI strains. Unfortunately, the RGP search engine looked at various signatures of DNA (GC skew, tRNA) but focused on specific regions within one genome compared to others, thus excluding a genomic island if shared by all (or almost all) genomes. We thus mapped our gene set to the HAIs detected by DNA signatures (GC skew, tRNAs, Insertion Sequences) in our previous study on ORS278 [13]. Twenty genes were found harbored by HAIs, and are labeled in bold in Table 3. Most genes belonged to two HAIs in ORS278 (BRADO5154-BRADO5204, and BRADO4807-4814), and were also located in HAI in all NI strains. These genes are involved in the biosynthesis of polysaccharides (EPS, LPS), a class of molecules known to play important roles in legume invasion during symbiosis (attachment to roots, biofilm formation, or interaction with plant defenses) [35].

To obtain further insight into the bacterial genes involved during the early steps of the Nod-independent symbiosis, a blind approach consisting in the screening of a large Tn5 mutant library (25,000 mutants) of the *Bradyrhizobium* ORS278 strain for their inability to induce nodules on *Aeschynomene* plants has been developed [12,13]. No strict nodulation-deficient mutants were found, but several mutants severely affected in nodule organogenesis were obtained. Among them, a mutant affected in a putative transcriptional factor of the TetR family (BRADO3272) was the only one to belong to the 446 NI specific gene pool. Interestingly, two upstream genes (BRADO3274 and BRADO3275) encode proteins of unknown functions and are also NI specific. Further studies are in progress in order to understand the role of this gene cluster during the NI symbiotic process. Apart from the BRADO3272 gene, no gene overlap could be found between mutants altered in symbiotic abilities and the comparative genomics approach. This result can be partially explained by the high redundancy of the 446 nod-independent specific gene set, of which 48% possess paralogs (>40% amino acid identity) in ORS278 (Supplementary Table S4). Symbiotic signals in classic rhizobia (Nod factors) and endomycorhizal fungi (Myc factors) are *N*-acetyl glucosamine derivatives [36,37,38]. Enzymes involved in Glucosamine modifications were detected in the 446 genes set, as BRADO6639, encoding an UDP-*N*-acetylglucosamine 1-carboxyvinyltransferase, and BRADO4235/BRADO7123, two homologs encoding a putative *O*-*N*-acetylglucosamine transferase related protein with TPR domains. These genes are also good candidates for future directed mutagenesis studies.

**Table 3:** List of specific annotated genes in nod-independent bradyrhizobia. Genes in bold are located in HAIs detected in ORS278.

CDS labels	Gene	Product
**Regulation**		
Two component regulatory systems		
**BRADO3895**		**putative two-component system, response regulator receiver**
BRADO4688		putative two-component system sensor protein with Hpt domain
BRADO4689		putative sensor histidine kinase with a response regulator receiver domain
BRADO5320		putative sensor histidine kinase (PAS & response regulator receiver)
BRADO6874		putative two component system, regulator receiver (CheY-like protein)
BRADO7009		putative response regulator receiver (CheY-like protein)
BRADO7010		putative sensor histidine kinase (receiver & phosphotransferase domains)
BRADO5682		putative two component sensor histidine kinase
BRADO1407		putative Two-component system histidine kinase
BRADO5100		Putative Two-component sensor histidine kinase
Transcriptional Regulators		
BRADO3272		putative transcriptional regulatory protein, TetR/AcrR family
BRADO3678		putative transcriptional regulatory protein, TetR family
BRADO5987		putative transcriptional regulator, TetR family
Cyclases		
BRADO0188		putative diguanylate cyclase (GGDEF) domain
BRADO1402		putative diguanylate cyclase (GGDEF)
BRADO2821		putative Adenylate cyclase with a CHAD domain
BRADO6510		putative diguanylate cyclase with GGDEF and EAL domains
Circadian clock operon		
BRADO1478	kaiC	circadian clock protein kinase kaiC
BRADO1479	kaiB	circadian clock protein
BRADO1480		putative signal transduction histidine kinase with PAS/PAC domains
BRADO1481		putative response regulator receiver (CheY-like protein)
**Catabolism**		
Plant wall degradation		
BRADO1851	vanB	vanillate *O* -demethylase oxidoreductase (Vanillate degradation)
BRADO2335-2343		protochatechuate transport and degradation (ligJABC)
BRADO2379		protocatechuate 4,5-dioxygenase (4,5-PCD), alpha chain
BRADO2380		2,3-dihydroxyphenylpropionate 1,2-dioxygenase
BRADO2382		putative transcriptional regulator, PadR-like family
BRADO4665	hpcB	homoprotocatechuate 2,3-dioxygenase
Various Catabolism/Detoxification		
BRADO0718		putative intradiol ring-cleavage dioxygenase
BRADO1842		putative amine oxidase
BRADO1843		putative ATPase, AAA family
BRADO1844		putative Glutathione S-transferase
BRADO1848		putative thiosulfate sulfurtransferase with Rhodanese-like domain
BRADO1849		conserved HP; putative oxidoreductase
BRADO4030		putative TauD/TfdA family dioxygenase (Taurine catabolism)
BRADO4031		putative dioxygenase; putative taurine dioxygenase (Taurine catabolism)
NUDIX hydrolase		
BRADO3028		putative MutT/nudix family protein
**BRADO3892**		**putative NUDIX-like hydrolase (modular protein)**
BRADO4664		putative NUDIX hydrolase
**Biosynthesis and modification enzymes**		
*N* -AcetylGlucosamine modifying enzymes		
BRADO3973		putative UDP- *N* -Acetylgucosamine-Peptide- *N* -Acetylglucosaminyl transferase subunit-related
BRADO4235		putative *O* -GlcNAc transferase related protein (TRP repeats)
BRADO6639		UDP- *N* -acetylglucosamine 1-carboxyvinyltransferase
BRADO7123		putative *O* -GlcNAc transferase related protein (TRP repeats)
Polysaccharide biosynthesis & glycans		
BRADO4794		putative D-3-phosphoglycerate dehydrogenase
BRADO4795		putative carbohydrate kinase (xylulose/erythritol kinase, lyx/eryA-like)
BRADO4796-4800		putative sugar ABC transporter (permease + ATP-binding)
BRADO4801		putative transcription regulator (EryD-like)
BRADO4802		putative carbohydrate kinase
BRADO4803		putative hydrolase (HAD superfamily)
BRADO4804		putative aldolase/epimerase (AraD-like)
**BRADO4807**		**putative 3-oxoacyl-acyl-carrier-protein reductase**
**BRADO4814**		**putative *N* -acetyl-mannosamine transferase (teich **
**BRADO5154**		**putative Capsular polysaccharide biosynthesis protein**
**BRADO5156**		**putative Asparagine synthetase**
**BRADO5164**		**putative bifunctional enzyme (sugar kinase/cytidylyltransferase)**
**BRADO5165**		**putative sugar-phosphate nucleotidyl transferase**
**BRADO5167**		**putative Phosphoheptose isomerase**
**BRADO5168**		**putative dTDP-glucose 4,6-dehydratase**
**BRADO5173**		**putative aminoglycoside phosphotransferase family protein**
**BRADO5197**		**putative Glycosyl transferase, group 1**
**BRADO5204**		**phosphoheptose isomerase (Sedoheptulose 7-phosphate isomerase)**
BRADO6336		putative NiFe-hydrogenase/urease accessory HupE/UreJ family protein
Others		
BRADO0151		putative Type III polyketide synthase; putative chalcone synthase
BRADO0860		putative asparagine synthetase (glutamine-hydrolyzing)
BRADO1346		putative Phenazine biosynthesis protein
BRADO1560		putative aldolase
BRADO4898		putative cobalt-precorrin-6A synthase, cbiD (Vitamin B12 biosynthesis)
BRADO4900		putative CobE protein (Vitamin B12 biosynthesis)
BRADO4906		putative precorrin-3B synthase (cobG) (Vitamin B12 biosynthesis)
BRADO4910		putative HupE/UreJ protein
BRADO4916		putative cobalamin synthase (CobS family) (Vitamin B12 biosynthesis)
BRADO4917		alpha-ribazole phosphatase (anaerobic pathway cobalamin biosynthesis)
BRADO7016		indole-3-pyruvate decarboxylase (IAA biosynthesis)
**Nitrogen fixation**		
BRADO5389	cysE	serine acetyltransferase
BRADO5390	nifV	homocitrate synthase
BRADO5396		putative transcriptional regulatory protein (protein cheY homolog)
BRADO5403		putative carboxymethylenebutenolidase(DLH)
BRADO5410		putative Ferredoxin, 2Fe-2S
BRADO5411		putative Aminotransferase, DegT/DnrJ/EryC1/StrS family
BRADO5414		conserved HP (NifZ domain)
BRADO5416		LRV protein FeS4 cluster in *N* -terminus
BRADO5417		conserved HP; TPR repeat
BRADO5418		putative iron-sulfur cluster assembly protein (SufA-like)
BRADO5424		putative nifU protein (C-terminal fragment)
**Carbon monoxide fixation**		
BRADO6025		putative Sensor histidine kinase (HWE family) with a GAF domain
BRADO6026	coxF	carbon monoxide dehydrogenase, coxF accessory protein
BRADO6027	coxE	carbon monoxide dehydrogenase, coxE accessory protein
BRADO6029	coxL	carbon monoxide dehydrogenase large chain
BRADO6031	coxM	carbon monoxide dehydrogenase medium chain (CO-DH M)
BRADO6032	coxC	carbon monoxide dehydrogenase, coxC signalling protein
**Photosynthesis & Carotenoid**		
BRADO0659-0662		carotenoid synthesis
BRADO2007		putative heme oxygenase
BRADO2008		putative bacteriophytochrome
**Quorum sensing**		
BRADO0941		autoinducer (acylhomoserine lactone) synthase
**Chemotaxis**		
BRADO2926		putative Methyl-accepting chemotaxis protein
BRADO0678		putative methyl-accepting chemotaxis protein
BRADO3031		putative methyl-accepting chemotaxis receptor/sensory transducer
BRADO4514		putative bacterial chemotaxis sensory transducer
BRADO5051		conserved HP-cheL
**Transport**		
Transporters		
BRADO0677		putative TRAP-dicarboxylate transporter (DctP subunit)
Secretion		
BRADO6338-6344		general secretion pathway proteins (GspI, GspL)
BRADO7122		putative HlyD-family secretion protein

The specific gene set of nod-dependent strains (Bj USDA110 and ORS285) was also investigated for functions of ecological interest. This gene set was 10 times smaller (49 genes) than the nod-independent one (446 genes). This low number of genes reflects the high phylogenetic distance between USDA110 (BJ clade) and ORS285 (BP clade), as all genes in PB have been subtracted from the gene pool of ORS285 in this comparative analysis. This result adds support to the hypothesis of a transfer of the symbiotic island from the BJ clade to BP. Interestingly, if such transfers can be detected, they are either rare since no other genes (apart from symbiotic ones) were detected in common, or they are very widespread in PB and so were present in the subtracted gene pool. Among the 49 common genes in nod-dependent strains, two main classes of genes with a predicted function can be distinguished: as expected, the *nod* genes (*nodD*, *nodA*, *nodB*, *nodC*, *nodS*, *nodU*, *nodI*, *nodJ*, *nodZ*, *noeI*, *nolA*), and more interestingly, the *rhc* genes (also named *tts*) that encode core components of a type three-secretion system (T3SS) [39,40].

The *nod* gene equipment therefore appears very similar between the two strains ORS285 and USDA110. The only differences observed are the additional presence in *B. japonicum* of: (i) *nolO* which encodes a carbamoyltransferase and (ii) the two component regulatory system *nodV/nodW.* In counter-part, ORS285 possesses an additional *nodL* gene that encodes an acetyl transferase. In accordance with similar *nod* genes content are the facts that both strains produce the same major Nod factor (a pentameric LCO with a 2-*O*-methylfucose at the reducing end) and are able to nodulate common host plants; in particular, the *B. japonicum* strain was recently shown to induce root and stem nodules on *A. afraspera* [41]. However, in contrast to the ORS285 strain, *B. japonicum* failed to induce nodule on *Aeschynomene* species which are nodulated by the nod-lacking strains such as *A. indica* and *A. sensitiva* suggesting that *B. japonicum* lacks key determinants required for the Nod-independent symbiotic process [34].

In *B. japonicum*, the *tts* gene cluster is found on a large symbiotic island of 681 kb [17]. As these genes are absent in the *nod-*lacking strains, we can postulate that ORS285 also acquired a symbiotic island harboring both the *nod* and the *tts* gene clusters by a lateral gene transfer. The T3SS is used by many bacterial pathogens to deliver directly a cocktail of effector proteins in eukaryotic cells that will subvert host defense [42]. This secretory machinery has been shown in several strains of rhizobia (including *B. japonicum* USDA 110) to affect symbiosis positively or negatively depending on the host [43]. It could therefore be particularly interesting to examine the impact of mutations in the *tts* cluster of the ORS285 strain to infer its role in the Nod-dependent or Nod-independent symbiotic processes in relation to the host plant.

## 3. Experimental Section

### 3.1. Bacterial Strains Maintenance and Preparation of Genomic DNA

Strains listed in Table 1 were grown on YMA [44], and bacterial genomic DNA was isolated following a modified CTAB protocol as recommended by the Joint Genome Institute [45].

### 3.2. Sequencing and Assembly of 7 Bradyrhizobium Genomes

Four genomes (ORS285, ORS375, STM3809, STM3843) were sequenced with Roche 454 GS-FLX technology (Genoscope, CEA, France) with a coverage of 11 to 28X, and with Illumina Solexa technology (GATC company, 36 bp-reads length, 20X cover). GS-FLX reads were assembled using Newbler software (v. 1.1.03.24), leading to 300 to 800 contigs. These contigs were fused to obtain a single scaffold, as a single chromosome and no plasmid could be visualized by PFGE analyses (not shown) and no *rep*C homologs could be found in the gene content of each of these genomes (*rep*C being a specific gene for plasmid replication in alphaproteobacteria). Solexa reads were used to correct contig consensus and sequencing errors. Three other genomes were sequenced only with Illumina Solexa technology (STM3847, STM4509 and STM4523) by the company GATC (Germany). Short 36 bp reads were pre-assembled with PERL script Vcake [46], using a first round of k-mer extensions of Solexa reads, then a second round using progressive extension of contigs obtained from the first round. This strategy allowed the construction of a sequence database of 100–140,000 contigs for each genome, subsequently used in global alignments. A last contig assembly was also built using CLC Genomics Workbench 4.0.2 (mismatch cost 2, Insertion cost 3, Deletion cost 3, length fraction 0.5, Similarity 0.8; length kept >50 bp), producing 12 to 14,000 contigs per strain, allowing gene search and phylogeny. Annotated genomes based on 454 GS-FLX assemblies were submitted to the EBI [47]. Solexa reads were submitted to the Sequence Read Archive (SAR) of the European Nucleotide Archive (ENA) under accession number ERP000868. Genomes are also available on the MicroScope annotation and comparative genomics platform [48] in the Rhizoscope Project.

### 3.3. Automatic and Expert Annotation of Bradyrhizobial Genomes

Annotation of bacterial genomes was conducted following 3 main steps: syntactic annotation to find genomic objects, followed by functional annotation to assign roles on the basis of sequence similarity with sequences from different databanks, and a relational annotation which establishes links between different genomic objects. All these steps were performed in the MicroScope platform pipeline and results are available via the MaGe (Magnifying Genomes) graphical interface [48,49]. For syntactic annotation, coding sequences were predicted using AMIGene (Annotation of Microbial Genomes) software [50]. Functional annotation used tools listed in [51] to give functions to the set of predicted genes. First, for strains ORS285, ORS375, STM3809 and STM3843, annotations were automatically transferred from strains ORS278, BTAi1 and *E. coli* K12 (in that order of priority) for genes having good similarities (*i.e*., 80% identity over at least 70% of the length of the smallest protein). These automatically generated results were stored in a relational database, and were further used as a starting point for manual validation with the MaGe web-interface, allowing data exploration, graphical visualizations and genomic comparisons. A functional classification was also produced for strain(-set)-specific genes using the KEGG database (via the KEGG Automatic Annotation Server [52]).

### 3.4. Whole Genome Alignments

Four sequenced *Bradyrhizobium* genomes were selected as reference. The remaining genomes were aligned on each reference genome using the MUMmer 3.0 whole genome global alignment system [53] to find conserved and organism-specific regions. In order to find potential alignment anchors, defined as maximal unique matches (MUMs) between two genome sequences, the MUMmer software uses suffix-trees. A suffix tree is a compact representation that stores all possible suffixes of an input sequence. MUMer then performed a protein alignment, starting by the translation of nucleic sequences in all 6 reading frames. Aligned regions between the reference and another genome that did not have a minimal identity of 70% and a match length of at least 20 amino-acids were filtered out. The CGview application [54] was used for graphical representation of whole genome alignments.

### 3.5. GC% Deviation

GC% deviation was computed using sliding 1,000 bp windows and represents the ratio between GC percent in the window and the mean GC percent of the genome. High deviations can indicate a potential foreign origin of the sequence within the window.

### 3.6. Core-Genome and Pangenome

Genes from two or more genomes were considered as orthologous if their encoded proteins satisfied bidirectional best-hit (BBH) criteria and alignment thresholds (40% amino acid identity on 80% of the length of the smallest protein). BBH are commonly used in comparative genomics to infer orthologous genes [55,56,57], and are integrated in the DOE IMG and MAGE platforms [49,58]. Two genes from two different genomes are bidirectional best hits when each is the best hit of the other. A bidirectional best-hit is considered as a good evidence that the genes may be orthologs arising from a common ancestor.

For Venn diagrams displayed in Figure 2 and Figure 6, comparison of gene sets between genomes were performed using the Phyloprofile Exploration tool of the MicroScope platform [48], using the following orthology criterion: BBH with 40% amino acid identity on 80% of the length of the smallest protein. Gene exploration in the 3 highly fragmented genomes (derived from solexa reads assemblies) was performed using tblastn searches with the Stand alone Blast software [59].

### 3.7. Phylogenomics

The core genome-based phylogeny was produced by aligning partitions for the 3,663 bradyrhizobia orthologs using ASAP software [60] (based on the Muscle alignment software [61]), and using PAUP4b10 to infer phylogenies on a combined partitions of all 3,663 genes (criterion set to distance, using Jukes-Cantor distance correction, and 100 bootstrap replicates). MLSA-based phylogeny shown in Figure 2B was obtained by Maximum Likelihood using a GTR + I + G model and 100 bootstrap replicates (using MEGA5 [62])

### 3.8. Search for Genomic Islands (GI)

The «RGPfinder» tool of the MicroScope annotation platform [63] is a comparative genomics method that searches for synteny breakpoints that delimit regions of at least 5 kbp in a target genome that are missing in one or more other related genomes. These RGPs (for Regions of Genomic Plasticity) can be insertion sites of GI, or the result of deletions of particular DNA segments in one or more strains. Region prediction is accompanied by the characterization of several common GI features, such as detection of tRNAs, IS (Insertion Sequence) and repeats, as well as information about nucleotide composition abnormalities [% (G + C) deviation, Codon Adaptation Index]. GI from two distinct organisms were considered identical when they shared homologous genes (at least 40% identity on 80% of the length of the smallest protein) in synteny on at least half of the GI (considering its size and number of genes). Correspondences between genomic islands in different *Bradyrhizobium* genomes were thus established in order to construct a binary matrix wherein each line represents a *Bradyrhizobium* genome and each column a GI. This matrix was then used to build a similarity tree which was compared to the phylogenetic tree of the *Bradyrhizobium* genomes.

### 3.9. Metabolic Comparisons

In each genome, the metabolic network was predicted by the “Pathway Tools” software [64] using MetaCyc [65] as a reference pathway database (version 13.0). The metabolic networks for each *Bradyrhizobium* sp. genome are directly available in the MaGe graphical interface at [66]. Using these data, a two-dimensional matrix was built for use in a Principal Component Analysis (with R package “FactoMineR”), which will highlight possible metabolic similarities and specificities between the genomes. For graphical representations, the variable (pathways) plot and the individual (genomes) plot were combined, restricting plotted variables to those with a quality of representation greater than 0.75, in order to conserve interpretability. The clustering method used a Euclidean distance and ward’s criterion; the number of classes was chosen after manual examination of the cluster tree, and led to 7 classes for the first factorial plan (see Supplementary Table S2).

## 4. Conclusions

Bacterial comparative genomics revealed a powerful tool to analyze *Aeschynomene* symbionts genome characteristics and their ecological niche adaptation. Photosynthetic *Bradyrhizobium* have large genomes (8–10 Mb), with a rather small core genome (3,663 genes) compared to their large pangenome (18,040 genes). The dispensable genome and strain-specific genes (mainly located on GI) exhibit numerous functions of adaptation towards the special ecological niche of PB (soil/aquatic, light-environment, stem/root nodule).

Our comparative genomics approach led us to the detection of specific gene pools in bradyrhizobial strains linked to two phenotypes of interest, *i.e.*, the photosynthetic ability and the nod-independent/nod-dependent symbiosis. Concerning bacterial photosynthesis, genome comparison reveals a clear ancestral origin for the photosynthetic activity. The LH2 type antennae complexes appear to be more widely distributed among PB that expected. The presence of a *cbb* gene cluster in synteny for all sequenced genomes suggests that preserving a functional CBB cycle confers a clear advantage. Finally, several new bacteriophytochromes were identified and further studies are required to explain their functional role. For the symbiotic process, we identified several target genes (as transcriptional regulators, polysaccharide biosynthesis and *N*-acetylglucosamine modification enzymes or plant-wall degrading enzymes) that will be further studied at the genetic level in order to prove their contribution to the nod-independent process. Finally, transcriptomic approaches on PB during their symbiosis with *Aeschynomeme* are currently being developed to identify more precisely the genetic determinants involved in this atypical mutualistic interaction.

## Supplementary Files

Supplementary File 1 ZIP-Document (ZIP, 11569 KB)

## References

[1] Gyaneshwar P., Hirsch A.M., Moulin L., Chen W.M., Elliott G.N., Bontemps C., Estrada de Los Santos P., Gross E., dos Reis F.B., Sprent J. (2011). Legume-nodulating betaproteobacteria: Diversity, host range and future prospects. Mol. Plant Microbe Interact..

[2] Nzoue A., Miche L., Klonowska A., Laguerre G., de Lajudie P., Moulin L. (2009). Multilocus sequence analysis of bradyrhizobia isolated from *Aeschynomene* species in Senegal. Syst. Appl. Microbiol..

[3] Rivas R., Martens M., de Lajudie P., Willems A. (2009). Multilocus sequence analysis of the genus *Bradyrhizobium*. Syst. Appl. Microbiol..

[4] James E.K., de Fatima Loureiro M., Pott A., Pott V.J., Martins C.M., Franco A.A., Sprent J.I. (2001). Flooding-tolerant legumes symbioses from the Brazilian Pantanal. New Phytol..

[5] Boivin C., Ndoye I., Lortet G., Ndiaye A., de Lajudie P., Dreyfus B. (1997). The sesbania root symbionts *Sinorhizobium saheli* and *S. teranga* bv. *sesbaniae* can form stem nodules on *Sesbania rostrata*, although they are less adapted to stem nodulation than *Azorhizobium* caulinodans. Appl. Environ. Microbiol..

[6] Bonaldi K., Gargani D., Prin Y., Fardoux J., Gully D., Nouwen N., Goormachtig S., Giraud E. (2011). Nodulation of *Aeschynomene afraspera* and *Aeschynomene indica* by photosynthetic *Bradyrhizobium* sp. strain ORS285: The Nod-dependent *versus* the Nod-independent symbiotic interaction. Mol. Plant Microbe Interact..

[7] Capoen W., Goormachtig S., Holsters M. (2010). Water-tolerant legume nodulation. J. Exp. Bot..

[8] D'Haeze W., Mergaert P., Prome J.C., Holsters M. (2000). Nod factor requirements for efficient stem and root nodulation of the tropical legume *Sesbania rostrata*. J. Biol. Chem..

[9] Evans W.R., Fleischman D.E., Calvert H.E., Pyati P.V., Alter G.M., Rao N.S. (1990). Bacteriochlorophyll and photosynthetic reaction centers in *Rhizobium* strain BTAi 1. Appl. Environ. Microbiol..

[10] Giraud E., Hannibal L., Fardoux J., Vermeglio A., Dreyfus B. (2000). Effect of *Bradyrhizobium* photosynthesis on stem nodulation of *Aeschynomene sensitiva*. Proc. Natl. Acad. Sci. USA.

[11] Giraud E., Fleischman D. (2004). Nitrogen-fixing symbiosis between photosynthetic bacteria and legumes. Photosynth. Res..

[12] Bonaldi K., Gourion B., Fardoux J., Hannibal L., Cartieaux F., Boursot M., Vallenet D., Chaintreuil C., Prin Y., Nouwen N. (2011). Large-scale transposon mutagenesis of photosynthetic *Bradyrhizobium* sp. strain ORS278 reveals new genetic loci putatively important for nod-independent symbiosis with *Aeschynomene indica*. Mol. Plant Microbe Interact..

[13] Giraud E., Moulin L., Vallenet D., Barbe V., Cytryn E., Avarre J.C., Jaubert M., Simon D., Cartieaux F., Prin Y. (2007). Legumes symbioses: Absence of Nod genes in photosynthetic bradyrhizobia. Science.

[14] Molouba F., Lorquin J., Willems A., Hoste B., Giraud E., Dreyfus B., Gillis M., de Lajudie P., Masson-Boivin C. (1999). Photosynthetic bradyrhizobia from *Aeschynomene* spp. are specific to stem-nodulated species and form a separate 16S ribosomal DNA restriction fragment length polymorphism group. Appl. Environ. Microbiol..

[15] Alazard D. (1985). Stem and root nodulation in *Aeschynomene* spp. Appl. Environ. Microbiol..

[16] Miche L., Moulin L., Chaintreuil C., Contreras-Jimenez J.L., Munive-Hernandez J.A., del Carmen Villegas-Hernandez M., Crozier F., Bena G. (2010). Diversity analyses of *Aeschynomene* symbionts in Tropical Africa and Central America reveal that nod-independent stem nodulation is not restricted to photosynthetic bradyrhizobia. Environ. Microbiol..

[17] Kaneko T., Nakamura Y., Sato S., Minamisawa K., Uchiumi T., Sasamoto S., Watanabe A., Idesawa K., Iriguchi M., Kawashima K. (2002). Complete genomic sequence of nitrogen-fixing symbiotic bacterium *Bradyrhizobium japonicum* USDA110. DNA Res..

[18] European Nucleotide Archive. http://www.ebi.ac.uk/ena/data/view/ERP000868/.

[19] Cytryn E.J., Jitacksorn S., Giraud E., Sadowsky M.J. (2008). Insights learned from pBTAi1, a 229-kb accessory plasmid from *Bradyrhizobium* sp. strain BTAi1 and prevalence of accessory plasmids in other *Bradyrhizobium* sp. strains. ISME J..

[20] Gil R., Silva F.J., Pereto J., Moya A. (2004). Determination of the core of a minimal bacterial gene set. Microbiol. Mol. Biol. Rev..

[21] Damerval T., Castets A.M., Guglielmi G., Houmard J., Tandeau de Marsac N. (1989). Occurrence and distribution of gas vesicle genes among cyanobacteria. J. Bacteriol..

[22] Buts L., Lah J., Dao-Thi M.H., Wyns L., Loris R. (2005). Toxin-antitoxin modules as bacterial metabolic stress managers. Trends Biochem. Sci..

[23] Oda Y., Larimer F.W., Chain P.S., Malfatti S., Shin M.V., Vergez L.M., Hauser L., Land M.L., Braatsch S., Beatty J.T. (2008). Multiple genome sequences reveal adaptations of a phototrophic bacterium to sediment microenvironments. Proc. Natl. Acad. Sci. USA.

[24] Giraud E., Fardoux J., Fourrier N., Hannibal L., Genty B., Bouyer P., Dreyfus B., Vermeglio A. (2002). Bacteriophytochrome controls photosystem synthesis in anoxygenic bacteria. Nature.

[25] Jaubert M., Zappa S., Fardoux J., Adriano J.M., Hannibal L., Elsen S., Lavergne J., Vermeglio A., Giraud E., Pignol D. (2004). Light and redox control of photosynthesis gene expression in *Bradyrhizobium*: Dual roles of two PpsR. J. Biol. Chem..

[26] Jaubert M., Vuillet L., Hannibal L., Adriano J.M., Fardoux J., Bouyer P., Bonaldi K., Fleischman D., Giraud E., Vermeglio A. (2008). Control of peripheral light-harvesting complex synthesis by a bacteriophytochrome in the aerobic photosynthetic bacterium *Bradyrhizobium* strain BTAi1. J. Bacteriol..

[27] Jaubert M., Lavergne J., Fardoux J., Hannibal L., Vuillet L., Adriano J.M., Bouyer P., Pignol D., Giraud E., Vermeglio A. (2007). A singular bacteriophytochrome acquired by lateral gene transfer. J. Biol. Chem..

[28] Lepo J.E., Hanus F.J., Evans H.J. (1980). Chemoautotrophic growth of hydrogen-uptake-positive strains of *Rhizobium japonicum*. J. Bacteriol..

[29] Gourion B., Delmotte N., Bonaldi K., Nouwen N., Vorholt J.A., Giraud E. (2011). Bacterial RuBisCO is required for efficient *Bradyrhizobium*/*Aeschynomene* symbiosis. PLoS ONE.

[30] Sudtachat N., Ito N., Itakura M., Masuda S., Eda S., Mitsui H., Kawaharada Y., Minamisawa K. (2009). Aerobic vanillate degradation and C1 compound metabolism in *Bradyrhizobium japonicum*. Appl. Environ. Microbiol..

[31] McLennan A.G. (2006). The Nudix hydrolase superfamily. Cell. Mol. Life Sci..

[32] Hakoyama T., Niimi K., Watanabe H., Tabata R., Matsubara J., Sato S., Nakamura Y., Tabata S., Jichun L., Matsumoto T. (2009). Host plant genome overcomes the lack of a bacterial gene for symbiotic nitrogen fixation. Nature.

[33] Ahlgren N.A., Harwood C.S., Schaefer A.L., Giraud E., Greenberg E.P. (2011). Aryl-homoserine lactone quorum sensing in stem-nodulating photosynthetic bradyrhizobia. Proc. Natl. Acad. Sci. USA.

[B34-genes-03-00035] 34.Giraud, E. LSTM, Montpellier, France. Unpublished work, 2011.

[35] Downie J.A. (2010). The roles of extracellular proteins, polysaccharides and signals in the interactions of rhizobia with legume roots. FEMS Microbiol. Rev..

[36] D'Haeze W., Holsters M. (2002). Nod factor structures, responses, and perception during initiation of nodule development. Glycobiology.

[37] Maillet F., Poinsot V., Andre O., Puech-Pages V., Haouy A., Gueunier M., Cromer L., Giraudet D., Formey D., Niebel A. (2011). Fungal lipochitooligosaccharide symbiotic signals in arbuscular mycorrhiza. Nature.

[38] Hamel L.P., Beaudoin N. (2010). Chitooligosaccharide sensing and downstream signaling: Contrasted outcomes in pathogenic and beneficial plant-microbe interactions. Planta.

[39] Kambara K., Ardissone S., Kobayashi H., Saad M.M., Schumpp O., Broughton W.J., Deakin W.J. (2009). Rhizobia utilize pathogen-like effector proteins during symbiosis. Mol. Microbiol..

[40] Krause A., Doerfel A., Gottfert M. (2002). Mutational and transcriptional analysis of the type III secretion system of *Bradyrhizobium japonicum*. Mol. Plant Microbe Interact..

[41] Renier A., Maillet F., Fardoux J., Poinsot V., Giraud E., Nouwen N. (2011). Photosynthetic *Bradyrhizobium* ORS285 synthesizes 2-*O*-methylfucosylated lipochitooligosaccharides for *nod* gene dependent interaction with *Aeschynomene* plants. Mol. Plant Microbe Interact..

[42] Hueck C.J. (1998). Type III protein secretion systems in bacterial pathogens of animals and plants. Microbiol. Mol. Biol. Rev..

[43] Wenzel M., Friedrich L., Gottfert M., Zehner S. (2010). The type III-secreted protein NopE1 affects symbiosis and exhibits a calcium-dependent autocleavage activity. Mol. Plant Microbe Interact..

[44] Vincent J.M. (1970). A Manual for the Pratical Study of Root-Nodule Bacteria. International Biological Programme Handbook.

[45] Joint Genome Institute http://my.jgi.doe.gov/general/.

[46] Jeck W.R., Reinhardt J.A., Baltrus D.A., Hickenbotham M.T., Magrini V., Mardis E.R., Dangl J.L., Jones C.D. (2007). Extending assembly of short DNA sequences to handle error. Bioinformatics.

[47] EMBL Nucleotide Sequence Database (EBI). http://www.ebi.ac.uk/genomes/.

[48] MAGE. MicroScope Annotation and Comparative Genomics Platform http://www.genoscope.cns.fr/agc/microscope/.

[49] Vallenet D., Labarre L., Rouy Z., Barbe V., Bocs S., Cruveiller S., Lajus A., Pascal G., Scarpelli C., Medigue C. (2006). MaGe: A microbial genome annotation system supported by synteny results. Nucleic Acids Res..

[50] Bocs S., Cruveiller S., Vallenet D., Nuel G., Medigue C. (2003). AMIGene: Annotation of microbial genes. Nucleic Acids Res..

[51] Vallenet D., Engelen S., Mornico D., Cruveiller S., Fleury L., Lajus A., Rouy Z., Roche D., Salvignol G., Scarpelli C. (2009). MicroScope: A platform for microbial genome annotation and comparative genomics. Database (Oxford).

[52] Moriya Y., Itoh M., Okuda S., Yoshizawa A.C., Kanehisa M. (2007). KAAS: An automatic genome annotation and pathway reconstruction server. Nucleic Acids Res..

[53] Kurtz S., Phillippy A., Delcher A.L., Smoot M., Shumway M., Antonescu C., Salzberg S.L. (2004). Versatile and open software for comparing large genomes. Genome Biol..

[54] Stothard P., Wishart D.S. (2005). Circular genome visualization and exploration using CGView. Bioinformatics.

[55] Li L., Stoeckert C.J., Roos D.S. (2003). OrthoMCL: Identification of ortholog groups for eukaryotic genomes. Genome Res..

[56] O'Brien K.P., Remm M., Sonnhammer E.L. (2005). Inparanoid: A comprehensive database of eukaryotic orthologs. Nucleic Acids Res..

[57] Overbeek R., Fonstein M., D'Souza M., Pusch G.D., Maltsev N. (1999). The use of gene clusters to infer functional coupling. Proc. Natl. Acad. Sci. USA.

[58] Mavromatis K., Chu K., Ivanova N., Hooper S.D., Markowitz V.M., Kyrpides N.C. (2009). Gene context analysis in the Integrated Microbial Genomes (IMG) data management system. PLoS ONE.

[59] Camacho C., Coulouris G., Avagyan V., Ma N., Papadopoulos J., Bealer K. (2009). BLAST+: Architecture and applications. BMC Bioinformatics.

[60] Sarkar I.N., Egan M.G., Coruzzi G., Lee E.K., DeSalle R. (2008). Automated simultaneous analysis phylogenetics (ASAP): An enabling tool for phlyogenomics. BMC Bioinformatics.

[61] Edgar R.C. (2004). MUSCLE: Multiple sequence alignment with high accuracy and high throughput. Nucleic Acids Res..

[62] Tamura K., Peterson D., Peterson N., Stecher G., Nei M., Kumar S. (2011). MEGA5: Molecular evolutionary genetics analysis using maximum likelihood, evolutionary distance, and maximum parsimony method. Mol. Biol. Evol..

[B63-genes-03-00035] 63.Roche, D. LABGeM, CEA Genoscope, Evry, France. Unpublished work, 2011.

[64] Karp P.D., Paley S., Romero P. (2002). The pathway tools software. Bioinformatics.

[65] Caspi R., Foerster H., Fulcher C.A., Kaipa P., Krummenacker M., Latendresse M., Paley S., Rhee S.Y., Shearer A.G., Tissier C. (2008). The MetaCyc Database of metabolic pathways and enzymes and the BioCyc collection of Pathway/Genome Databases. Nucleic Acids Res..

[66] Microcyc. https://www.genoscope.cns.fr/agc/microscope/metabolism/microcyc.php/.

