# Supplementary material for: Comparative Genomics of Aeschynomene Symbionts: Insights into the Ecological Lifestyle of Nod-Independent Photosynthetic Bradyrhizobia
Source: Genes (Basel). 2011 Dec 21;3(1):35–61. doi: 10.3390/genes3010035 (PMC3899966; doi:10.3390/genes3010035)
Supplement: Supplementary File 1 — ZIP-Document (ZIP, 11569 KB) [file genes-03-00035-s001.zip › genes-11761-Supplementary Material/genes-11761-Supplementary Figures.doc]

Supplementary Figures

**Figure S1.** Relative synonymous codons usage frequencies in *Bradyrhizobium*.

**Figure S2.** Functional distribution of core, dispensable and specific genes of bradyrhizobia.

**Figure S3.** KEGG functional annotation of group of genes (strain/NI/ND/PB specific).

**Figure S4.** Whole genome comparisons of bradyrhizobia.

**Figure S5.** Figure S5 A&B: Metabolic network comparison of strains.

**Figure S1.** Relative synonymous codons usage frequencies in *Bradyrhizobium* sp. ORS278. All PB strains exhibited the same codon usage.

**Figure S2.** Functional distribution of core, dispensable and specific genes of bradyrhizobia (ORS278, BTAi1, ORS285, STM3809, ORS375, STM3843, USDA110). Gene function was attributed using the KEGG protein classification (via the KEGG Automatic Annotation Server-KAAS at <http://www.genome.jp/tools/kaas/>). (**A**) % of conserved/hypothetical/protein with annotated function; (**B**) KEGG classification in percentage of total of strain-specific, dispensable and core functionally annotated genes; (**C**) Same as B, but classification is based on gene numbers, not percentages.

C

**Figure S3.** KEGG functional annotation of group of genes strain-specific, dispensable or core, or specific of photosynthetic/ non-photosynthetic strains, and use of
nod-dependent/nod-independent symbiotic pathways.

**Figure S4.** Whole genome comparisons of bradyrhizobia (using Mummer). See Figure 2 legend for details.

**Figure S4.** *Cont*.

**Figure S4.** *Cont*.

**Figure S4.** *Cont*.

**Figure S4.** *Cont*.

**Figure S4.** *Cont*.

**Figure S5.** Principal Component Analysis of seven *Bradyrhizobium* genomes, performed from a two dimensional matrix, combining genomes and metabolic pathways.

(**A**)

**Figure S5.** *Cont*.

(**B**)

Individual points represent genomes, and colored vectors symbolize completion of pathways (number of reaction for pathway x in a given organism/total number of reactions in the same pathway x defined in the MetaCyc database) in the data (see method section). Pathways with similar completions (vectors with similar orientation) have been clustered and drawn in a same color. Thus, genomes can be associated with their representative and characteristic groups of metabolic pathways (*i.e.*, vectors pointing in their direction). The corresponding pathway functions are listed in Supplementary Table S3.
